# Variants in the CxxC domain of the epigenetic regulator *KDM2B* support its role in developmental eye anomalies

**DOI:** 10.1038/s41431-026-02090-1

**Published:** 2026-04-07

**Authors:** Fabiola Ceroni, Linda M. Reis, Fiona Watkins, Dorine A. Bax, Megan C. Fischer, Karthikah Jeganathan, Rosalyn Jewell, Jacob S. Martin, Alison Salt, Sarah E. Seese, Jenny Thomson, Elena V. Semina, Nicola K. Ragge

**Affiliations:** 1 School of Biological and Medical Sciences, Faculty of Health, Science and Technology, Oxford Brookes University, Oxford, UK; 2 Department of Ophthalmology and Visual Sciences, Medical College of Wisconsin, Milwaukee, WI USA; 3 Leeds Clinical Genomics Service, Chapel Allerton Hospital, Leeds Teaching Hospitals NHS Trust, Leeds, UK; 4 Kids Rehab WA, Perth Children’s Hospital, Nedlands, WA Australia; 5 UCL, Great Ormond Street Institute of Child Health, London, UK; 6 Department of Pediatrics and Children’s Research Institute, Medical College of Wisconsin and Children’s Wisconsin, Milwaukee, WI USA; 7 Department of Cell Biology, Neurobiology and Anatomy, Medical College of Wisconsin, Milwaukee, WI USA; 8 West Midlands Regional Clinical Genetics Service, Birmingham Women’s and Children’s NHS Foundation Trust and Birmingham Health Partners, Birmingham, UK

**Keywords:** Genetics research, Genetic testing, Clinical epigenetics

## Abstract

*KDM2B* encodes an epigenetic regulator that binds to promoter-associated CpG islands *via* its CxxC zinc-finger domain, protecting them from DNA methylation. It also helps establish transcriptional programs essential for development by recruiting the non-canonical Polycomb Repressive Complex 1.1 to lineage-specific genes. Heterozygous variants in *KDM2B* were recently associated with a neurodevelopmental disorder. Notably, some individuals with variants in the CxxC domain also exhibited congenital heart, kidney and/or structural eye anomalies. By screening 706 families with developmental eye disorders, we identified two cases with *KDM2B*-CxxC variants, NM_032590.5:c.1841G>C;p.(Arg614Pro) and NM_032590.5:c.1880G>C;p.(Cys627Ser), both resulting in a characteristic *KDM2B* DNA episignature. Both individuals exhibited complex structural eye defects, with neurodevelopmental, cardiac and renal anomalies variably present. These cases strengthen the association between *KDM2B*-CxxC variants and eye, kidney and heart malformations and highlight the importance of testing this gene and its episignature in individuals with structural eye disorders, especially when accompanied by congenital cardiac and/or renal anomalies.

## Introduction

Structural eye anomalies, including anophthalmia, microphthalmia and coloboma, are genetically heterogeneous disorders, occurring as part of identifiable syndromes in 20–45% of cases [1]. Over 140 genes are currently tested on diagnostic panels, including the UK PanelApp ‘Structural Eye Disease’ (https://panelapp.genomicsengland.co.uk/panels/509/).

Mendelian Disorders of the Epigenetic Machinery (MDEMs) are an expanding group of conditions caused by variants in epigenetic regulators, including *CHD7* (CHARGE syndrome, OMIM: 214800) and *KMT2D* (Kabuki syndrome 1, OMIM: 147920). Recently, monoallelic variants in *KDM2B* (*Lysine demethylase 2B*) were associated with a new MDEM presenting with developmental delay (DD), variable systemic features and a characteristic DNA methylation profile (‘*KDM2B-*associated episignature’) [2]. To date, three 12q24.31 deletions and 22 pathogenic/potentially pathogenic single-nucleotide variants (SNVs) have been reported in 28 families [2–4]. Of these, 17 different SNVs clustered within the small zinc-finger domain CxxC [5, 6]. Intriguingly, *KDM2B*-CxxC variants generated a specific ‘sub-episignature’, suggesting a distinct molecular effect [2]. Compared with individuals carrying non-CxxC variants, CxxC cases additionally presented with congenital heart defects (17/21) and kidney agenesis (6/21). Over half (13/21) had ophthalmological findings; while these mainly included strabismus, myopia and astigmatism, 3/13 individuals exhibited severe structural eye anomalies (not observed in non-CxxC cases), suggesting that *KDM2B*-CxxC variants influence eye morphogenesis.

To gain further insight into this, we analysed 706 families with developmental eye anomalies, identifying two new cases with pathogenic *KDM2B*-CxxC missense variants. Both displayed complex structural eye disorders and extra-ocular features consistent with those previously reported in *KDM2B*-CxxC cases.

## Subjects and methods

Families 1 and 2 were identified through whole exome/genome sequencing (WES/WGS) analysis of 706 families with structural eye anomalies (456/706 genetically undiagnosed).

Family 1: WES of proband and maternal DNA was performed by Psomagen (Rockville, US-MD) [7] and SNVs and copy number variants (CNVs) analysed using VarSeq (Golden Helix, Bozeman, US-MT) [8].

Family 2: WGS of proband and parental DNA was performed by Theragen Bio (Republic of Korea). SNVs were analysed using an in-house pipeline prioritising rare coding and canonical splice variants. CNVs and structural variants were assessed using Optical Genome Mapping (Bionano Genomics, US-CA).

DNA methylation profiles were assessed using the EpiSign assay [9, 10]. *KDM2B* variants were validated using Sanger sequencing. Pathogenicity was ascribed using ACMG/AMP criteria following the UK-ACGS guidelines [11, 12].

## Results

### Family 1

**Fig. 1:**
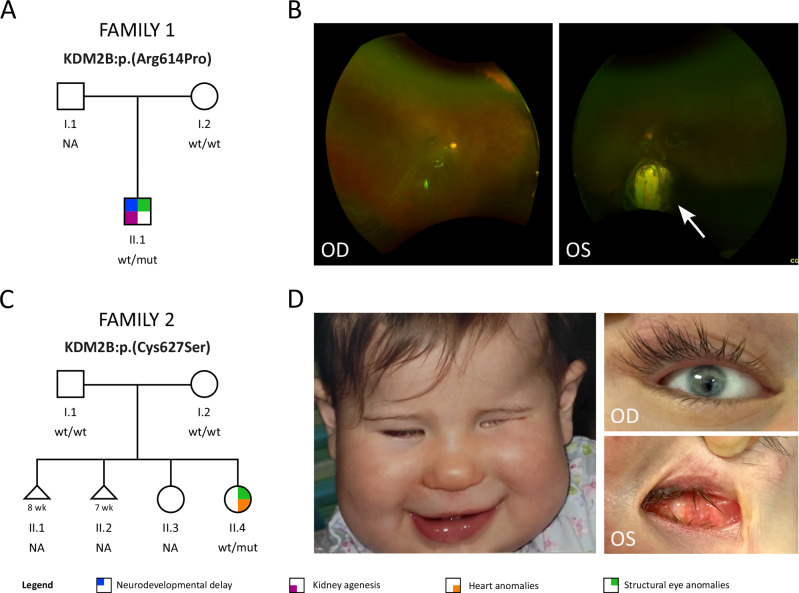
Novel families with pathogenic *KDM2B* variants and structural eye anomalies. **A** Pedigree of Family 1. The proband (II.1) carries the *KDM2B* variant c.1841G>C;p.(Arg614Pro), absent in the mother (I.2). Paternal DNA was not available (NA). **B** Ocular images of Proband II.1 (Family 1). Right eye (OD) shows an increased cup to disc ratio (0.80); left eye (OS) shows an increased cup to disc ratio (0.65) and coloboma inferior to the optic nerve (arrow). **C** Pedigree of Family 2. The proband (II.4) carries the de novo *KDM2B* variant c.1880G>C;p.(Cys627Ser). Her older sister is unaffected. Triangles indicate miscarriages (*wk* weeks of gestation). **D** Photographs of Proband II.4 (Family 2). Left panel: image of the proband as a baby showing bitemporal narrowing, straight eyebrows, underdeveloped left socket (left vestigial eye remnant), right microphthalmia, smooth philtrum, thin upper lip and fleshy lower lip. Right panel: images of the proband’s eyes at 18 years of age. Right eye (OD): mild microphthalmia with disorganised anterior segment; left eye (OS): anophthalmia.

Case 1 (Individual II.1, Fig. 1A) is a 9-year-old black male, born at full-term by normal delivery (birth weight: 3.15 kg). He was diagnosed with bilateral congenital glaucoma (buphthalmos). He underwent right trabeculotomy and trabeculectomy at 14 days of age and Ahmed glaucoma valve (AGV) insertion at 1 month of age (right) and 2 months of age (left). At 9 years of age he exhibited right exotropia, bilateral myopia with buphthalmos, bilateral iris hypoplasia with corectopia, left posterior embryotoxon, a small left anterior capsule cataract (detected at 8 months of age and possibly AGV-related) and a small left chorioretinal coloboma (Fig. 1B). He displayed facial asymmetry, high forehead, bitemporal narrowing, malar hypoplasia, small ears and short stature (8 years of age: 117.2 cm; just <3^rd^ centile). He had generalized delayed dental development and required removal of two persistent primary teeth at 8 years of age; at 9 years of age, he had partially erupted molars and persistent primary incisors. He also had DD with possible autism spectrum disorder.

Chromosomal microarray revealed a 1q21.3 deletion (chr1:152,028,001-152,250,046, hg38), not detected in maternal WES data. This was deemed non-contributory, since there are no haploinsufficient genes within the region. WES identified a heterozygous *KDM2B* missense variant [NM_032590.5:c.1841G>C;p.(Arg614Pro)], absent from gnomAD v4.1.0. The change was not detected in the unaffected mother; paternal DNA was unavailable. The variant, located within the CxxC domain, is predicted to be damaging/probably damaging by multiple in silico tools (dbNSFP v5.3, https://www.dbnsfp.org/). The same change is also reported in ClinVar (SCV005079302; phenotype unavailable) and in a published case [4]; an additional case displayed a different amino acid change at Arg614 [NM_032590.5:c.1841G>T;p.(Arg614Leu)] [3]. While previously published cases with Arg614 variants did not undergo DNA methylation testing, EpiSign analysis of Case 1 detected the *KDM2B*-associated episignature with ‘moderate’ confidence. The variant was classified as pathogenic (Table 1).

**Table 1:** Phenotypic and genetic findings of the two cases presented in this study.

	Case 1	Case 2
Sex	male	female
Age at last evaluation	9 years	18 years
**Clinical findings**		
**Postnatal development**		
Micro/macrocephaly	unknown	microcephaly (<3^rd^ centile from 17 mo)
Short stature	yes (<3^rd^ centile at 8 years)	no (25^th^ centile at 18 years)
Developmental delay	yes	mild motor delay (rolled over: 6-7 mo, sat unsupported: 8-9 mo, walked: 17-21 mo), no speech delay (single words: 16 mo)
Behavioural disorders	possible autism spectrum disorder (not formally assessed)	no
**Facial dysmorphisms**		
High forehead	yes	no
Facial asymmetry	yes	no
Bitemporal narrowing	yes	yes
Malar hypoplasia	yes	no
Microtia	yes	no
**Eye anomalies**		
Anophthalmia/microphthalmia	no	yes (R microphthalmia, L clinical anophthalmia)
Anterior segment anomalies	yes (BL iris hypoplasia with corectopia, L posterior embryotoxon)	yes (disorganised R anterior segment with small iris coloboma/corectopia)
Chorioretinal coloboma	yes (L)	unknown
Optic nerve aplasia	no	yes (BL)
Hypoplasia of the optic tract	unknown (no MRI)	yes (BL)
Primary congenital glaucoma	yes (BL)	no
Buphthalmos	yes (BL, R > L)	no
Exotropia	yes (R)	no
Myopia	yes (R: –8.25 D, L: –0.50 D)	N/A (no light perception)
**Other features**		
Congenital kidney anomalies	yes (USS: L kidney agenesis)	no (USS: normal)
Congenital heart anomalies	no	yes (ASD)
Abnormality of dental eruption	yes	yes
Joint hypermobility (lax ligaments)	no	yes
***KDM2B*** **variant**		
Genomic position (hg38)	chr12:121,453,238 (C > G)	chr12:121,453,199 (C > G)
Coding effect (NM_032590.5)	c.1841G>C; p.(Arg614Pro)	c.1880G>C; p.(Cys627Ser)
Protein domain [PM1]	CxxC	CxxC (key residue for Zinc binding)
Inheritance mode [PS2]	not maternal, father unavailable	de novo
gnomAD (v4.1.0) [PM2]	absent	absent
Same/different changes at the same residue [PS1/PM5]	c.1841G>C; p.(Arg614Pro) de novo (ref. [4]) c.1841G>T; p.(Arg614Leu) de novo(ref. [3])	c.1880G>A; p.(Cys627Tyr) de novo (ref. [2])
In silico predictions [PP3]		
SIFT (score)	damaging (0.001)	damaging (0)
Polyphen2 HVAR (score)	probably damaging (0.977)	probably damaging (0.997)
AlphaMissense (score)	likely pathogenic (0.9993)	likely pathogenic (0.9999)
CADD_phred score	34	32
REVEL score	0.421	0.824
*KDM2B* episignature [PP4]	yes (moderate confidence)	yes (strong confidence)
ACMG/AMP criteria applied	PS1, PM1_supporting (critical domain), PM2, PM5, PP2, PP3, PP4	PS2, PM1_moderate (critical domain + key residue), PM2, PM5, PP2, PP3, PP4
ACMG/AMP classification	pathogenic	pathogenic

Variant pathogenicity was classified according to the UK-ACGS guidelines, using the following criteria: PS1: same missense change previously reported as pathogenic; PS2: de novo; PM1: located in a functional domain and/or a key residue with no benign variation, PM2: absent from controls (gnomAD v4.1.0); PM5: different missense change at the same amino acid residue reported as pathogenic; PP2: gene where missense variants are a common disease mechanism (*KDM2B* constraint to missense variation: *Z* = 4.63, gnomAD v4.1.0); PP3: Multiple lines of computational evidence support a deleterious effect (in silico predictions were retrieved from dbNSFP v5.3); PP4: *KDM2B* episignature.

*ASD* atrial septal defect, *BL* bilateral, *D* dioptres, *L* left, *mo* months-of-age, *MRI* magnetic resonance imaging scan, *N/A* not applicable, *R* right, *USS* ultrasound scan.

Following genetic diagnosis, renal ultrasound identified a solitary right kidney, while echocardiography was normal.

### Family 2

Case 2 (Individual II.4, Fig. 1C) is a 19-year-old female, born at 41^+ 6^ weeks’ gestation by normal delivery after induction, following a pregnancy complicated by gestational diabetes. Her birth weight was 4.45 kg (90–97^th^ centile), head circumference (HC) on the 50^th^ centile. At one week of age, she was diagnosed with left anophthalmia (3.5 mm anophthalmic remnant) and mild right microphthalmia (16 mm axial length) (Fig. 1D). Visual evoked potentials were absent on the left and probably absent on the right. Sequential left socket expansion using hydrophilic expanders was commenced. At 5 months of age, MRI revealed delayed myelination and a 3 mm intradiploic epidermoid cyst midline in the frontal bone, while confirming a left anophthalmic remnant with reduced orbital size and right microphthalmia. Optic nerves and chiasm were not demonstrable; optic tracts were hypoplastic. She had mildly delayed motor development (she rolled over at 6–7 months of age, sat unsupported at 8–9 months of age, never crawled and walked at 17–21 months of age), although this progression was within the normal range for a child with visual impairment. She had no speech delay, cognitive impairment or behavioural issues. At 17 months of age, her HC had fallen to <2^nd^ centile (length: 50^th^ centile, weight: 9^th^ centile). Echocardiography revealed an isolated ostium secundum atrial septal defect (ASD) surgically closed at 3 years 4 months. Teeth were generally crowded; both upper canines failed to erupt normally and had been removed. Other features included bitemporal narrowing and hypermobility. Renal ultrasound confirmed normal kidneys. At 18 years of age, she exhibited microcephaly (HC: 52.3 cm [0.4^th^–2^nd^ centile], height: 160 cm [25^th^ centile], weight: 48.5 kg [9^th^ centile]), left anophthalmia, right microphthalmia with a disorganised anterior segment and a small iris coloboma/corectopia (Fig. 1D) and no light perception in either eye.

Case 2’s WGS identified a de novo *KDM2B* missense variant [NM_032590.5:c.1880G>C;p.(Cys627Ser)], absent from gnomAD v4.1.0. The change, predicted to be damaging by multiple in silico algorithms (dbNSFP v5.3), affects a CxxC residue directly involved in Zn^2+^ binding [2]. A different variant affecting Cys627 [NM_032590.5:c.1880G>A;p.(Cys627Tyr)] was previously reported as pathogenic [2, 3]. Case 2 tested positive for the *KDM2B*-associated episignature with ‘strong’ confidence (EpiSign v5). The variant was classified as pathogenic (Table 1). No other SNVs/CNVs of relevance to her eye phenotype were identified.

## Discussion

We describe two individuals with *KDM2B* variants [p.(Arg614Pro) and p.(Cys627Ser)] manifesting significant developmental eye anomalies and variable neurodevelopmental, renal and cardiac phenotypes.

**Fig. 2:**
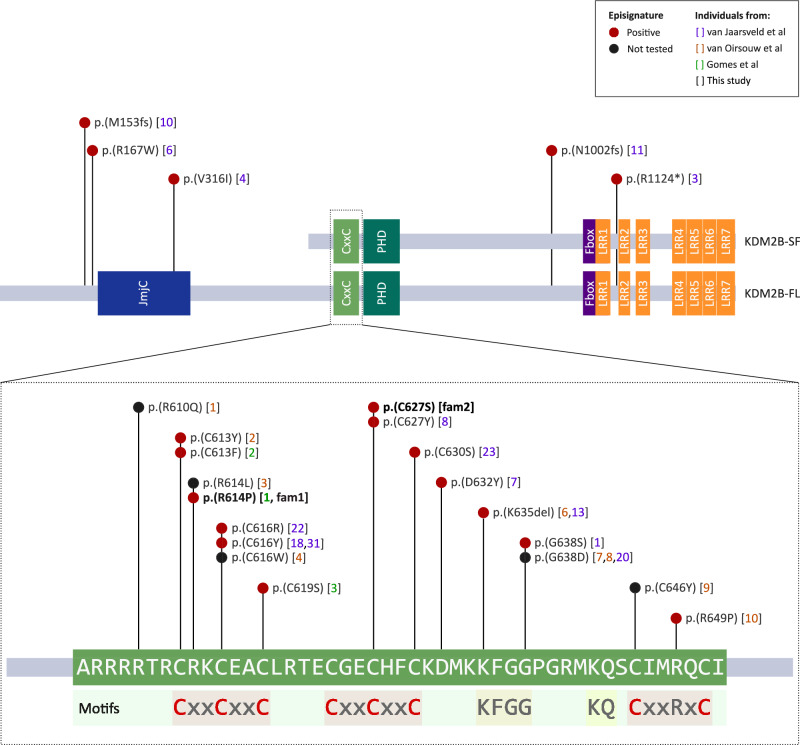
Schematic of KDM2B with pathogenic variants reported in this and previous studies. Protein domains are reported according to UniProt entry Q8NHM5. Both the short (SF) and the full-length (FL) isoforms contain a DNA-binding domain (CxxC), a plant homeodomain (PHD), an F-box and a Leucine-rich repeat (LRR). The amino acids forming the CxxC zinc-finger domain are indicated with the single-letter amino acid code in the bottom panel. The CxxC domain contains two cysteine-rich clusters (CxxCxxCx_4_CGxCxxC and CxxRxC), with eight cysteines (indicated in red) coordinating two Zn^2+^ ions in a tetrahedral manner and a linker region containing the highly conserved motif KFGG (Lys-Phe-Gly-Gly) and the DNA-binding motif KQ (Lys-Gln) [5]. The latter, together with the upstream residue (Met), directly interacts with the CpG dinucleotide. The two variants identified in this study are indicated in bold, together with pathogenic variants from previous studies [2–4]. Family identifiers (square brackets) are reported according to the numbers used in previous publications: van Jaarsveld et al. [2] in purple, van Oirsouw et al. [3] in orange, Gomes et al. [4] in green, this study in black. Red dots indicate variants where at least one case exhibited the *KDM2B* episignature; black dots indicate variants for which methylation analyses were not performed.

*KDM2B* has two main isoforms, both containing a CxxC domain, a plant homeodomain (PHD), an F-box and a Leucine-rich repeat (LRR). The full-length isoform additionally includes a JmjC histone demethylase domain (Fig. 2). KDM2B binds promoter-associated unmethylated CpG islands *via* CxxC [6, 13], protecting them from de novo methylation [14, 15]. Moreover, in mouse embryonic stem cells (mESCs), Kdm2b recruits the non-canonical Polycomb Repressive Complex 1.1 to early lineage gene promoters, helping establish epigenetic transcriptional programmes essential for development [6,13].

*KDM2B* variants have recently been implicated in a new MDEM, with CxxC emerging as a hotspot domain [2–4]. In addition to speech delay (21/21), motor delay (18/21), intellectual disability (ID) or learning difficulties (10/13), behavioural issues (9/19), microcephaly (4/18) or macrocephaly (2/18), brain (7/12) and skeletal anomalies (14/21), CxxC cases also exhibited congenital heart (17/21), kidney (6/21) and structural eye anomalies (3/21) (Supplementary Table).

By screening families recruited for structural eye anomalies, we identified two new cases with *KDM2B*-CxxC variants. Case 1 [p.(Arg614Pro)] displayed bilateral iris hypoplasia with corectopia, left posterior embryotoxon and chorioretinal coloboma, left kidney agenesis, facial asymmetry, small ears, short stature, DD and possible autism. A 2-year-old boy with the same variant [p.(Arg614Pro) [4]] showed solitary kidney, cardiac anomalies, myopic astigmatism, plagiocephaly, facial asymmetry and global DD. A 15-year-old boy carrying the variant p.(Arg614Leu) [3] exhibited simple ears, short stature, speech and motor delay, severe ID, behavioural disorders, but no eye anomalies. Case 2 [p.(Cys627Ser)] had left anophthalmia, right microphthalmia with anterior chamber anomalies, apparent optic nerve aplasia, microcephaly, ASD, but no kidney anomalies. A previously described individual carrying the variant p.(Cys627Tyr) [2] had cardiac defects, kidney agenesis, short stature, mild DD, behavioural issues and normal eyes, suggesting that congenital anomalies also occur variably with Cys627 variants. Importantly, Case 2 had mild early motor delay attributable to her visual impairment, but no speech, cognitive or behavioural issues. Both Case 1 and 2 had anomalous dental development (persistent primary dentition and/or delayed tooth eruption), also described in another CxxC case [p.(Arg649Pro) [3]], possibly expanding the *KDM2B* spectrum. Interestingly, dental, cardiac and structural eye anomalies also occur in individuals with variants in *BCOR* (OMIM: 300485) [16], a PRC1.1 component interacting with KDM2B.

Cases 1 and 2 tested positive for the *KDM2B*-associated episignature, supporting the deleterious role of these variants. Case 1’s episignature was called with moderate confidence, potentially reflecting a hypomorphic effect of p.(Arg614Pro) [10]. Unlike Cys627, Arg614 is not directly involved in Zinc binding; Arg614 variants were predicted to affect local protein structure instead [3]. However, as seen for other recurrent *KDM2B*-CxxC variants, Case 1 and the previously described individual with p.(Arg614Pro) displayed different heart and eye phenotypes, suggesting that modifying factors (genetic, environmental and/or stochastic) also contribute to the variable expressivity of congenital anomalies. Given *KDM2B*’s widespread role in gene repression [17], variants in target genes or other developmental regulators of gene expression may also influence the phenotypic outcome.

Mouse models support the importance of *Kdm2b* in the heart, eye and neuro-development. Homozygous deletions disrupting both *Kdm2b* isoforms are embryonically lethal, causing severe malformations, including neural tube, craniofacial and cardiovascular defects [14, 18]. Deletion of the *Kdm2b*-CxxC domain (*Kdm2b*^Δ*CxxC*^) also led to semi-lethality and congenital anomalies in heterozygosity [19]. While ocular phenotypes were not assessed in *Kdm2b*^Δ*CxxC*^ animals, ~40% of mouse embryos lacking full-length *Kdm2b* displayed neural tube defects, retinal coloboma and expanded neuroretina, highlighting *Kdm2b*’s involvement in eye development [20].

In summary, our study supports the role of *KDM2B*-CxxC dysfunction in heart, kidney and eye malformations and emphasises the importance of testing *KDM2B* and its episignature in individuals with structural eye anomalies, particularly when renal and/or cardiac findings are present.

### Web resources

ClinVar: https://www.ncbi.nlm.nih.gov/clinvar/. dbNSFP: https://www.dbnsfp.org/. gnomAD: https://gnomad.broadinstitute.org/. OMIM: https://www.omim.org/. PanelApp: https://panelapp.genomicsengland.co.uk/panels/509/. UniProt: https://www.uniprot.org/.

## Supplementary information

Supplementary Table

## Data Availability

The variants identified in this study were submitted to ClinVar (Accession IDs: SCV005889720; SCV005687775).

## References

[1] Slavotinek A. Genetics of anophthalmia and microphthalmia. Part 2: syndromes associated with anophthalmia-microphthalmia. Hum Genet. 2019;138:831–46. 30374660 10.1007/s00439-018-1949-1

[2] van Jaarsveld RH, Reilly J, Cornips MC, Hadders MA, Agolini E, Ahimaz P, et al. Delineation of a *KDM2B*-related neurodevelopmental disorder and its associated DNA methylation signature. Genet Med. 2023;25:49–62. 36322151 10.1016/j.gim.2022.09.006 PMC9825659

[3] van Oirsouw ASE, Hadders MA, Koetsier M, Peters EDJ, Assia Batzir N, Barakat TS, et al. KDM2B variants in the CxxC domain impair its DNA-binding ability and cause a distinct neurodevelopmental syndrome. Hum Mol Genet. 2025;34:1353–67. 10.1093/hmg/ddaf082 PMC12361114 40420380

[4] Gomes A, Martín-Rodríguez Á, Del Campo M, Bird LM. *KDM2B*-related neurodevelopmental disorder: a case-series supporting the CxxC domain phenotype with emphasis on ocular and dermatologic features. Am J Med Genet A. 2025. 10.1002/ajmga.70036 (online ahead of print). 10.1002/ajmga.70036 41457890

[5] Long HK, Blackledge NP, Klose RJ. ZF-CxxC domain-containing proteins, CpG islands and the chromatin connection. Biochem Soc Trans. 2013;41:727–40. 23697932 10.1042/BST20130028 PMC3685328

[6] Farcas AM, Blackledge NP, Sudbery I, Long HK, McGouran JF, Rose NR, et al. KDM2B links the polycomb repressive complex 1 (PRC1) to recognition of CpG islands. Elife. 2012;1:e00205. 23256043 10.7554/eLife.00205 PMC3524939

[7] Reis LM, Atilla H, Kannu P, Schneider A, Thompson S, Bardakjian T, et al. Distinct roles of histone lysine demethylases and methyltransferases in developmental eye disease. Genes. 2023;14:216. 10.3390/genes14010216 PMC9859058 36672956

[8] Iacocca MA, Wang J, Dron JS, Robinson JF, McIntyre AD, Cao H, et al. Use of next-generation sequencing to detect *LDLR* gene copy number variation in familial hypercholesterolemia. J Lipid Res. 2017;58:2202–9. 28874442 10.1194/jlr.D079301 PMC5665663

[9] Aref-Eshghi E, Kerkhof J, Pedro VP, Barat-Houari M, Ruiz-Pallares N, Andrau JC, et al. Evaluation of DNA methylation episignatures for diagnosis and phenotype correlations in 42 Mendelian neurodevelopmental disorders. Am J Hum Genet. 2020;106:356–70. 32109418 10.1016/j.ajhg.2020.01.019 PMC7058829

[10] Kerkhof J, Rastin C, Levy MA, Relator R, McConkey H, Demain L, et al. Diagnostic utility and reporting recommendations for clinical DNA methylation episignature testing in genetically undiagnosed rare diseases. Genet Med. 2024;26:101075. 38251460 10.1016/j.gim.2024.101075

[11] Richards S, Aziz N, Bale S, Bick D, Das S, Gastier-Foster J, et al. Standards and guidelines for the interpretation of sequence variants: a joint consensus recommendation of the American College of Medical Genetics and Genomics and the Association for Molecular Pathology. Genet Med. 2015;17:405–24. 25741868 10.1038/gim.2015.30 PMC4544753

[12] Durkie M, Cassidy E, Berry I, Owens M, Turnbull C, Scott RH, et al. ACGS Best practice guidelines for variant classification in rare disease 2024. United Kingdom: Association for Clinical Genomic Science (ACGS). Available from: https://www.genomicseducation.hee.nhs.uk/wp-content/uploads/2024/08/ACGS-2024_UK-practice-guidelines-for-variant-classification.pdf.

[13] He J, Shen L, Wan M, Taranova O, Wu H, Zhang Y. Kdm2b maintains murine embryonic stem cell status by recruiting PRC1 complex to CpG islands of developmental genes. Nat Cell Biol. 2013;15:373–84. 23502314 10.1038/ncb2702 PMC4078788

[14] Boulard M, Edwards JR, Bestor TH. FBXL10 protects Polycomb-bound genes from hypermethylation. Nat Genet. 2015;47:479–85. 25848754 10.1038/ng.3272

[15] Kawamura YK, Ozonov EA, Papasaikas P, Kondo T, Nguyen NV, Stadler MB, et al. Preventing CpG hypermethylation in oocytes safeguards mouse development. Dev Cell. 2025;60:3285–303.e9. 10.1016/j.devcel.2025.08.005 PMC12687725 40902605

[16] Ragge N, Isidor B, Bitoun P, Odent S, Giurgea I, Cogné B, et al. Expanding the phenotype of the X-linked *BCOR* microphthalmia syndromes. Hum Genet. 2019;138:1051–69. 29974297 10.1007/s00439-018-1896-x

[17] Turberfield AH, Kondo T, Nakayama M, Koseki Y, King HW, Koseki H, et al. KDM2 proteins constrain transcription from CpG island gene promoters independently of their histone demethylase activity. Nucleic Acids Res. 2019;47:9005–23. 31363749 10.1093/nar/gkz607 PMC6753492

[18] Andricovich J, Kai Y, Peng W, Foudi A, Tzatsos A. Histone demethylase KDM2B regulates lineage commitment in normal and malignant hematopoiesis. J Clin Investig. 2016;126:905–20. 26808549 10.1172/JCI84014 PMC4767361

[19] Blackledge NP, Farcas AM, Kondo T, King HW, McGouran JF, Hanssen LLP, et al. Variant PRC1 complex-dependent H2A ubiquitylation drives PRC2 recruitment and polycomb domain formation. Cell. 2014;157:1445–59. 24856970 10.1016/j.cell.2014.05.004 PMC4048464

[20] Fukuda T, Tokunaga A, Sakamoto R, Yoshida N. Fbxl10/Kdm2b deficiency accelerates neural progenitor cell death and leads to exencephaly. Mol Cell Neurosci. 2011;46:614–24. 21220025 10.1016/j.mcn.2011.01.001

